# Strengthening National Immunization Technical Advisory Groups: Twelve Years of Progress (2012–2023)

**DOI:** 10.3390/vaccines13010080

**Published:** 2025-01-17

**Authors:** Louise Henaff, Laure Dumolard, Vinod Bura, Gerald Etapelong Sume, Sidy Ndiaye, Jennifer Sanwogou, Heeyoun Cho, Joachim Hombach, Christoph A. Steffen

**Affiliations:** 1 World Health Organization (WHO), 1211 Geneva, Switzerland; dumolardl@who.int (L.D.); hombachj@who.int (J.H.); steffenc@who.int (C.A.S.); 2 World Health Organization (WHO) Regional Office for South-East Asia, New Delhi 110002, India; burav@who.int; 3 World Health Organization (WHO) Regional Office for the Eastern Mediterranean, Cairo 11371, Egypt; sumeetapelongg@who.int; 4 World Health Organization (WHO) Regional Office for Africa, Brazzaville P.O. Box 06, Congo; ndiayes@who.int; 5 World Health Organization (WHO) Regional Office for the Americas, Washington, DC 20037, USA; sanwogoj@paho.org; 6 World Health Organization (WHO) Regional Office for the Western Pacific, Manila 1000, Philippines; hcho@who.int

**Keywords:** National Immunization Technical Advisory Groups, NITAGs, immunization policy, WHO–UNICEF Joint Reporting Form

## Abstract

Introduction: Well-functioning National Immunization Technical Advisory Groups (NITAGs) are valuable contributors to decision-making processes in the complex immunization policy arena. This paper describes the progress made globally on the establishment and strengthening of these key advisory groups and discusses some of their strengths, challenges, and opportunities. Methods: The data submitted annually by countries to the World Health Organization (WHO) via the WHO/UNICEF Joint Reporting Form (JRF) were analyzed, comparing the NITAG functionality criteria in 2012 and 2023. Results: In 2023 and 2012, 88% and 61% of countries, respectively, reported having a NITAG. A total of 77% of NITAGs met all six NITAG process criteria in 2023 compared to 33% in 2012. This progress was most notable in the WHO African Region, increasing from 7% (2012) to 77% (2023), and the South-East Asia Region, increasing from 45% (2012) to 91% (2023). In 2023, 84% of NITAGs issued a vaccine-policy recommendation that was adopted by decision-makers. Discussion: Marked progress has been made since 2012 on establishing and maintaining NITAGs, with a small number of countries yet to form an advisory committee. Supporting and sustaining NITAG functions remains an important means for countries to foster independent and transparent expert advice on vaccine and immunization policy. Setbacks in countries facing instability or political turmoil are a reminder of the reversibility of progress. WHO and partners play an important role in supporting countries in strengthening these advisory committees. Continuous commitment by countries to the function and involvement of NITAGs in policy recommendations is essential for enhancing the strength and resilience of immunization programs.

## 1. Introduction

National Immunization Technical Advisory Groups (NITAGs) are pivotal to vaccine and immunization policy processes in many countries, providing independent, transparent, and evidence-based advice to national immunization policymakers. Decisions to introduce new vaccines into a country’s immunization program result in long-term financial and programmatic commitments and establish community expectations. Beyond advising on vaccine introductions, NITAGs also guide policymakers on optimizing existing immunization programs and addressing broader health system challenges, such as low vaccine coverage and programmatic barriers. The advice from NITAGs is also important for longer-term immunization program planning, such as that defined in the National Immunization Strategy (see the following: https://www.who.int/teams/immunization-vaccines-and-biologicals/vaccine-access/planning-and-financing/nis, accessed on 1 October 2024). Hence, through providing independent expert reviews and advice, NITAGs have become increasingly important in guiding and advising decision-makers.

The state of NITAGs at the global and regional levels in 2012 has been previously published [1]. The aim of this paper is to describe the global and regional progress on NITAG development and strengthening over the past 12 years. To this effect, this paper presents a global overview of NITAGs in 2023, comparing it to the situation in 2012 through an analysis of NITAG indicators reported via the WHO–UNICEF Joint Reporting Form on Immunization (JRF).

## 2. Background

The first NITAGs, established in the 1960s, included the United Kingdom’s Joint Committee on Vaccination and Immunisation (JCVI) (1963); the United States’ Advisory Committee on Immunization Practices (ACIP) (1964); and Canada’s National Advisory Committee on Immunization (NACI) (1964). These advisory committees marked the beginning of formalized bodies providing independent, expert advice on vaccine and immunization policy.

The Agence de Médecine Préventive launched the Supporting Independent Immunization and Vaccine Advisory Committees (SIVAC) Initiative in 2008. This WHO Collaborating Center, funded by the Bill & Melinda Gates Foundation and Gavi, helped establish and strengthen more than 20 NITAGs across Africa, Asia, and Eastern Europe over a period of 10 years [2].

To better understand the progress in NITAG development globally, WHO added specific questions about their functionality to the JRF on vaccines and immunization in 2010. These additions allowed for the annual monitoring of NITAG progress and facilitated advocacy for establishing NITAGs in countries that lacked them. Data from the JRF enabled targeted capacity-building efforts and the provision of tailored technical support.

The Global Vaccine Action Plan (GVAP), endorsed by the World Health Assembly in 2012, was pivotal in advocating for the scale-up and strengthening of NITAGs globally. GVAP emphasized that every country should have access to the services of a functional NITAG and urged governments not only to establish these committees but also to equip them to support informed, independent, evidence-based decision-making regarding vaccines [3].

When the SIVAC initiative ended in 2017, WHO and technical partners swiftly transitioned the support function by assigning dedicated staff at WHO headquarters and regional offices and building regional partnerships with various institutions, such as the NITAG Support Hub (NISH), founded in 2021 and based in Cape Town, South Africa, to ensure continued support for NITAGs globally. Regional partners play a key role in assisting WHO in the training of newly formed NITAGs.

The Immunization Agenda 2030, which details the vaccination and immunization strategic priorities for 2030, reaffirms the importance of functional NITAGs for the success of immunization programs worldwide. Building on the momentum created by GVAP, the agenda identifies NITAGs as a critical component for ensuring the sustainability of immunization policies, country ownership, and the introduction of new vaccines [4].

## 3. Methods

Data for this paper were compiled from the 2012 and 2023 WHO–UNICEF JRFs. This standard questionnaire—sent annually to all Member States since 1998—collects data on all immunization aspects, including vaccine coverage, reported cases of vaccine-preventable diseases, immunization schedules, as well as performance indicators of the immunization program and financing. Questions relating to NITAGs in the JRF included a set of six process indicators pertaining to the characteristics and functioning of a NITAG [5]. These indicators are as follows: (i) a legislative or administrative basis for the advisory group; (ii) formal written terms of reference; (iii) a diversity of expertise among core members (referencing pediatrics, public health, infectious diseases, epidemiology, immunology, or other relevant areas of health-care expertise); (iv) the number of meetings held per year (a minimum of one meeting per year is needed to meet the requirement of this indicator); (v) circulation of the agenda and background documents at least one week prior to meetings; and (vi) the mandatory disclosure of any conflicts of interest.

In addition to these process-related indicators, WHO added in 2021 two output indicators to the JRF questionnaire: (i) did the NITAG issue one or more recommendations (during the year)?; and (ii) are one or more of the NITAG recommendations adopted by the Ministry of Health (during the year)?

More specific information on the JRF and the data collection process is published on the WHO website [6].

The results were stratified by WHO Regions, World Bank national income status categories [7], eligibility for support by Gavi [8] (which includes all countries with a gross national income of ≤USD 1810 per capita in accordance with World Bank data for the latest available year), and population size for the year 2023. Population figures used are those from the United Nations Population Division [9].

Note that all 2012 comparator figures are taken from the 2013 published analysis and are not included in the visuals of this paper.

## 4. Results

The 2023 status of the NITAG-related indicators at global and regional levels is presented in Table 1, while Table 2 provides an analysis of these indicators, stratified by World Bank income groups, eligibility for GAVI support, and population size.

NITAG-related JRF data were available from 193 of the 194 WHO Member States in 2023, compared with 191 Member States in 2012. With the exception of the Cook Islands, which did not report in 2023, all Member States reporting data in 2012 also reported data in 2023. Countries that did not report in 2012 but did so in 2023 include Austria, Monaco, and Serbia.

In 2023, 88% (170/193) of reporting countries, representing 98% of the global population, reported having a NITAG to guide their immunization policy decisions. By comparison, in 2012, only 61% (116/191) of reporting countries, covering 89% of the global population, reported having a NITAG (Table 1).

When examining countries that reported having a NITAG that met all six process criteria, the progress between the two periods is even more apparent. In 2023, 68% of reporting countries (131/193), representing 84% of the global population, had a NITAG meeting all six criteria. In contrast, in 2012, only 33% of countries, covering 52% of the global population, had a NITAG that met these criteria.

Progress varied across WHO Regions. The African Region saw an increase in countries meeting all six criteria, with 77% (36/47 countries, covering 98% of the population) in 2023 up from 7% of countries (3/46 countries representing 7% of the region’s population) in 2012. Similarly, in the South-East Asia Region, 91% (10/11) of countries in 2023 had a NITAG meeting the six criteria, while only 45% (5/11) of countries had the same in 2012.

In the Region of the Americas and the European Region, the increase was also noticeable. In 2023, NITAGs that met the six criteria were reported in 74% (26/35) and 74% (39/53) of countries, respectively. In 2012 this was the case for only 37% (13/35) and 44% (22/50) of reporting countries, respectively.

In the Western Pacific Region, progress had stalled, with 50% (13/26) of countries reporting the existence of a NITAG in 2023 versus 56% (15/27) in 2012. Likewise, 23% (6/26) of NITAGs reported meeting all six process criteria in 2023, versus 26% (7/27) in 2012. This was due largely to the absence of progress in small Pacific Island countries.

In the Eastern Mediterranean Region, the proportion of countries meeting the six criteria increased slightly, rising to 67% (14/21) of countries in 2023 compared to 62% (13/21) in 2012.

The process criteria with the lowest scores in 2023 were “circulation of the agenda and background documents ahead of the meetings” and “holding at least one NITAG meeting per year”. In contrast, in 2012, “mandatory disclosure of conflicts of interest” obtained the lowest achievement rate.

As shown in Table 2, the breakdown of these indicators by income class for 2023 shows that low- and high-income countries reported similar proportions of NITAG existence (94% and 96%, respectively). Middle-income countries reported a slightly lower proportion, with 84% having an established NITAG. However, when considering the proportion of NITAGs meeting all six process criteria, low-income countries led, with 84% meeting the criteria, while high- and middle-income countries reported lower scores of 69% and 64%, respectively. This is a notable shift from the pattern observed in 2012, when high-income countries consistently outperformed middle- and low-income countries in both the existence of NITAGs and meeting the six process criteria. Moreover, larger countries with populations above 10 million showed a higher proportion of reporting countries that met the six process criteria (74%) compared to smaller countries (61%).

**Figure 1:**
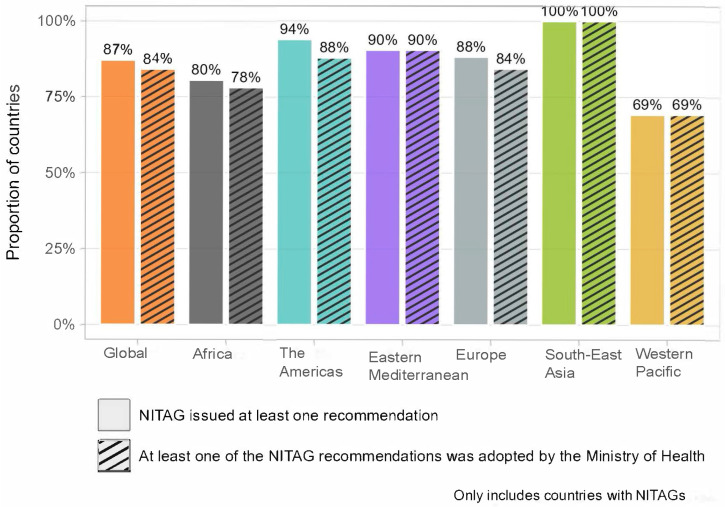
Proportion of countries with a NITAG that, in 2023, (i) had a NITAG issuing recommendations, and (ii) had NITAG recommendations adopted by the Ministry of Health, by WHO Region.

The NITAG output indicators provide further insight into the performance of NITAGs (Figure 1). Globally, in 2023, 87% of countries with a NITAG reported issuing recommendations in the past year, with 84% having the recommendations adopted by their Ministry of Health. The regional analysis, however, reveals disparities. All countries in the South-East Asia Region reported issuing NITAG recommendations in 2023 that were adopted by their Ministry of Health, fully meeting all indicators for the NITAG. By contrast, in the Western Pacific Region, only 69% of countries issued recommendations that had been adopted in 2023. The other four WHO Regions (Africa, the Americas, Europe, and Eastern Mediterranean) displayed relatively high performance for both indicators, ranging from 78% to 94%.

**Figure 2:**
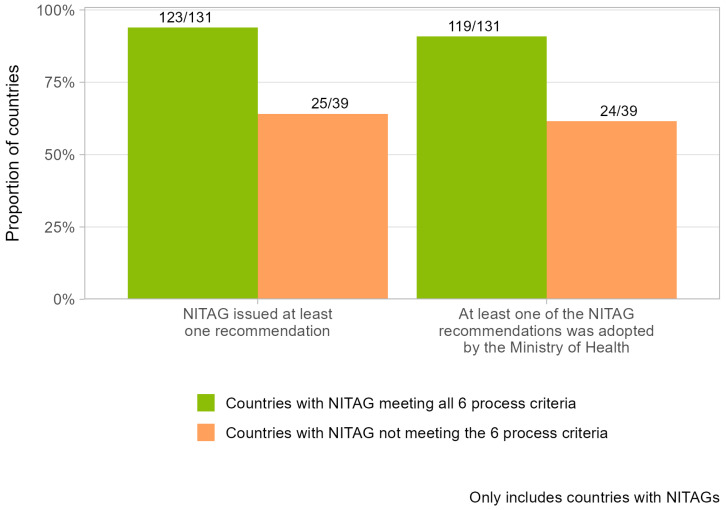
Proportion of countries with a NITAG that, in 2023 (i) had a NITAG issuing recommendations, and (ii) had NITAG recommendations adopted by the Ministry of Health, by their process-criteria status.

The ability of NITAGs to issue recommendations adopted by the Ministry of Health varied according to the functionality status of the NITAGs (Figure 2). Globally, 94% of countries with a NITAG that met the six process criteria issued at least one recommendation in 2023, compared with 64% of countries that did not meet those criteria. Likewise, 91% of countries with NITAGs meeting the six process criteria had at least one NITAG recommendation adopted by their Ministry of Health in 2023, compared with 62% of countries that did not meet those criteria.

## 5. Discussion

The formation and functionality of NITAGs improved significantly between 2012 and 2023. During this period, global and regional stakeholders supported countries in establishing NITAGs and strengthening their capacities to carry out their important advisory role in supporting decision-makers.

Efforts have resulted in significant progress in the African Region and to a lesser degree also in the Eastern Mediterranean Region [10]. In 2023 alone, the WHO African Regional Office provided training courses for nine NITAGs on their role, responsibilities, and the evidence-to-recommendation framework, while the Eastern Mediterranean Regional Office conducted similar training for 10 countries.

In the South-East Asia Region, the progress on NITAG functionality has, likewise, been notable. The relatively small number of countries (with very large populations) facilitated more targeted support. For example, the South-East Asia Regional Office conducted independent external evaluations of NITAGs in the region during 2019–2020, enabling each NITAG to develop a clear improvement plan.

The finding that low-income countries currently have a higher proportion of NITAGs that meet all six process criteria is testament to the commitment and support these countries have mobilized, including from WHO and other partners, to strengthen their capabilities. Low-income countries also benefit from Gavi financial support, which enables the establishment of technical secretariats; the organization of meetings; the participation of NITAG members in international meetings; and NITAG-strengthening activities.

The number of NITAGs reported globally—currently in 170 of 194 countries—demonstrates how even small nations and territories have succeeded in building mechanisms to access the expertise of an advisory group. A notable example is in the Caribbean, where the first subregional Caribbean Immunization Technical Advisory Group was formed, serving 22 countries and territories. This collaborative approach highlights the creativity, adaptability, and innovation of small countries in ensuring that they are part of global immunization policy efforts [11].

The Pacific Island countries, some with populations of just tens of thousands and often scattered over wide territories, are also exploring ways to pool resources to support subregional evidence-based decision-making. The lack of a tailored solution for Pacific Island countries has contributed to the delay in forming NITAGs in the Western Pacific Region.

The fact that larger countries seem to perform somewhat better than smaller countries on the six process criteria suggests that there may be a size threshold under which it becomes increasingly difficult for small countries to have a functional committee. Pooling resources within or between countries is even more important in those settings.

The circulation of the agenda and background documents at least one week prior to meetings serves as an indicator of the ability of the NITAG secretariats to function effectively. In 2023, this was the weakest indicator, suggesting a bottleneck for many NITAGs. Indeed, without dedicated secretariat resources, the sustainability and effectiveness of NITAG operations cannot be guaranteed. Currently, there is no clear definition of “an adequately resourced technical secretariat”, and the JRF does not include specific questions regarding this. However, it is increasingly evident that the absence of dedicated technical staff to manage secretariat functions leads to delays in the work of NITAGs and weakens the evidence-based recommendation process. One option to document this could be to add to the JRF an additional question regarding the existence of a dedicated technical NITAG secretariat, accompanied by a clear definition of its role and responsibilities. Reporting on this additional criterion could help programs advocate more successfully for dedicated funding and staff for the secretariat.

Over three-quarters of countries with functioning NITAGs issued at least one recommendation in 2023, most of which were adopted by their respective ministries of health. This is a relatively crude but simple way to measure the output of NITAGs annually. However, it does not allow for the drawing of granular conclusions on differences in the reported figures on those indicators. The slightly higher score on the issuance of recommendations versus the adoption by the Ministry of Health is consistent with the fact that NITAGs are advisory bodies and that other considerations come into play in the decision-making process, such as budgetary and programmatic considerations. Moreover, the finding that countries that meet the six process criteria perform better overall on these outcomes supports the approach of WHO and partners to continue strengthening NITAG secretariats.

## 6. Actionable Recommendations

The Global NITAG Network (GNN) has called for a detailed analysis of instances where NITAG recommendations have not been adopted by ministries of health. Understanding the delays in implementing recommendations is crucial as they may stem from various factors, including budgetary constraints, logistical challenges, competing (health) priorities, or the need for further alignment with national or subnational policies or contexts. Further investigation is needed to identify specific barriers and to support the development of strategies that facilitate faster adoption and implementation of NITAG recommendations, ensuring timely improvements in public health outcomes. Moreover, ensuring that NITAGs also consider programmatic and economic considerations in their evidence-to-recommendation process will contribute to making recommendations more fit for purpose and applicable for governments.

NITAGs operating in politically unstable contexts face unique challenges in maintaining functionality and ensuring evidence-based decision-making for immunization. In such environments, political turmoil, conflicts, and frequent changes in government can disrupt the consistency of NITAG meetings and hinder the implementation of their recommendations—although this is not fully evident in the 2023 JRF data alone. NITAGs that had existed for several years are being dismantled by changes in government, while others, barely set up, see their work abruptly interrupted. Further analysis is needed to capture the status and challenges of NITAGs operating in these situations.

In fragile settings, often with concomitant health emergencies, NITAGs could be instrumental in addressing critical programmatic issues, such as the maintenance of cold chains, ensuring vaccination coverage in remote or conflict-affected areas, and responding to outbreaks amidst challenging conditions. Flexibility in the response is paramount, and NITAGs could play an important role in this regard. The Regional Immunization Technical Advisory Group of the Eastern Mediterranean Region called for further documentation from countries to fully capture the role of NITAGs in these volatile environments and to explore strategies that could ensure their continuity during political transitions or instability. Strengthening support mechanisms and safeguarding their operations, even amidst governmental changes, are essential for preserving the ability of NITAGs to provide informed, evidence-based decisions for national immunization programs.

The JRF questionnaire covers a wide range of immunization-related topics, limiting the opportunity to include additional questions regarding NITAGs. The current NITAG-related questions do not sufficiently capture the complexity of their functionality nor reflect the nuances of their role in national policymaking. These limitations hinder a comprehensive assessment of how well NITAGs are integrated into the broader immunization decision-making system at the country level. WHO, therefore, encourages NITAGs to regularly evaluate their committees using a standardized assessment process, which would help identify gaps and promote long-term sustainability. The NITAG Maturity Assessment Tool (NMAT) developed by the United States Centers for Disease Control and Prevention, WHO, and other partners at the request of the GNN, provides a systematic evaluation framework and offers clear recommendations for strengthening NITAG functionality. The NMAT also enables NITAGs to measure their progress over time, ensuring continuous improvement and alignment with global standards [12].

## 7. Conclusions

Between 2012 and 2023, NITAGs increasingly became the global standard for providing expert advice to governments on immunization policies. Functionality, as measured by the six process criteria, progressed substantially, particularly in low- and middle-income countries. With most of the world’s population now covered by a NITAG, it is crucial for countries to focus on sustaining the functionality of these committees. Looking ahead, sustained support for NITAG technical secretariats and the enhanced use of assessment tools—such as the NITAG Maturity Assessment Tool—will be key to further improving their functionality and effectiveness, enabling them to provide independent, evidence-based, and transparent immunization policy guidance.

## Figures and Tables

**Table 1:** Analysis of the NITAG 2024 Joint Reporting Form data for 2023, globally and by WHO Region.

Countries with NITAG Data Available/WHO Member States	Indicator	Region						
		OVERALL N = 193/194 (99%)	AFR N = 47/47 (100%)	AMR N = 35/35 (100%)	EMR N = 21/21 (100%)	EUR N = 53/53 (100%)	SEAR N = 11/11 (100%)	WPR N = 26/27 (96%) ^a^
Existence of a NITAG	Number of countries	170	41	33	21	51	11	13
	% of countries with NITAG data available	88%	87%	94%	100%	96%	100%	50%
	% of the entire population covered	98%	99%	96%	100%	99%	100%	93%
Existence of a NITAG with formal terms of reference	Number of countries	168	40	33	21	50	11	13
	% of countries reporting the existence of a NITAG	99%	98%	100%	100%	98%	100%	100%
	% of the entire population covered	97%	98%	96%	100%	94%	100%	93%
Existence of a NITAG with a legislative or administrative basis	Number of countries	168	39	33	21	51	11	13
	% of countries reporting the existence of a NITAG	99%	95%	100%	100%	100%	100%	100%
	% of the entire population covered	97%	98%	96%	100%	99%	100%	93%
Existence of a NITAG with ≥5 areas of expertise represented	Number of countries	162	40	30	19	50	10	13
	% of countries reporting the existence of a NITAG	95%	98%	91%	90%	98%	91%	100%
	% of the entire population covered	96%	99%	93%	96%	99%	99%	93%
Existence of a NITAG which met at least once in 2023	Number of countries	153	39	31	19	46	11	7
	% of countries reporting the existence of a NITAG	90%	95%	94%	90%	90%	100%	54%
	% of the entire population covered	91%	98%	83%	91%	83%	100%	83%
Existence of a NITAG for which the agenda and background documents distributed ≥1 week prior to meetings	Number of countries	153	39	31	19	46	11	7
	% of countries reporting the existence of a NITAG	90%	95%	94%	90%	90%	100%	54%
	% of the entire population covered	91%	98%	83%	91%	83%	100%	83%
Existence of a NITAG whose members are required to disclose conflicts of interest	Number of countries	168	39	31	16	45	11	12
	% of countries reporting the existence of a NITAG	99%	95%	94%	76%	88%	100%	92%
	% of the entire population covered	97%	98%	75%	87%	90%	100%	92%
Existence of a NITAG meeting all 6 criteria above	Number of countries	131	36	26	14	39	10	6
	% of reporting countries	68%	77%	74%	67%	74%	91%	23%
	% of countries reporting the existence of a NITAG	77%	88%	79%	67%	76%	91%	46%
	% of the entire population covered	84%	98%	59%	79%	73%	99%	81%

WHO Regions: AFR: African Region; AMR: Region of the Americas; EMR: Eastern Mediterranean Region; EUR: European Region; SEAR: South-East Asia Region; WPR: Western Pacific Region. ^a^ The only WHO Member State for which data are missing is the Cook Islands. N = total number of countries that reported in 2024 on NITAG data for 2023.

**Table 2:** Analysis of the NITAG 2024 Joint Reporting Form data for 2023, by World Bank income status, Gavi eligibility, and population size.

	World Bank Income Status ^a^			Gavi-Eligible Countries	Population Size	
	Low Income	Middle Income	High Income		Less than 9,193,372 ^b^	Greater than or Equal to 9,193,372
Reporting Countries/Total (%)	26/26 (100%)	103/103 (100%)	62/62 (100%)	57/57 (100%)	96/97 (99%)	97/97 (100%)
NITAG exists/No. Reporting (%)	25/26 (96%)	87/103 (84%)	58/62 (94%)	52/57 (91%)	78/96 (81%)	92/97 (95%)
Formal terms of reference/No. Reporting (%)	25/26 (96%)	86/103 (83%)	57/62 (92%)	51/57 (89%)	77/96 (80%)	91/97 (94%)
Legislative or administrative basis for NITAG/No. Reporting (%)	25/26 (96%)	85/103 (83%)	58/62 (94%)	51/57 (89%)	76/96 (79%)	92/97 (95%)
At least 5 areas of expertise represented/No. Reporting (%)	24/26 (92%)	82/103 (80%)	56/62 (90%)	50/57 (88%)	75/96 (78%)	87/97 (90%)
Met at least once in 2023/No. Reporting (%)	23/26 (88%)	80/103 (78%)	50/62 (81%)	47/57 (82%)	71/96 (74%)	82/97 (85%)
Agenda and background documents distributed ≥1 week prior to meetings/No. Reporting (%)	23/26 (88%)	80/103 (78%)	50/62 (81%)	47/57 (82%)	71/96 (74%)	82/97 (85%)
Required to disclose conflicts of interest/No. Reporting (%)	23/26 (88%)	78/103 (76%)	53/62 (85%)	48/57 (84%)	69/96 (72%)	85/97 (88%)
Meeting all 6 criteria above/No. Reporting (%)	22/26 (85%)	66/103 (64%)	43/62 (69%)	43/57 (75%)	59/96 (61%)	72/97 (74%)

^a^ The denominator is not the 194 WHO Member States but 191 countries for which the World Bank is providing the information, as of July 2024. Information on income status is missing for the Cook Islands, Niue, and Venezuela (the Bolivarian Republic of). ^b^ This figure was selected as being the median of the total population for the 194 WHO Member States in 2023.

## Data Availability

Data used in this analysis are officially reported by WHO Member States through the electronic Joint Reporting Form (https://www.who.int/teams/immunization-vaccines-and-biologicals/immunization-analysis-and-insights/global-monitoring/who-unicef-joint-reporting-process/, accessed 1 October 2024), and publicly available from the WHO Immunization Data Portal (https://immunizationdata.who.int/, accessed 1 October 2024).
